# The Flavonoid Luteolin, but Not Luteolin-7-*O*-Glucoside, Prevents a Transthyretin Mediated Toxic Response

**DOI:** 10.1371/journal.pone.0128222

**Published:** 2015-05-28

**Authors:** Irina Iakovleva, Afshan Begum, Malgorzata Pokrzywa, Malin Walfridsson, A. Elisabeth Sauer-Eriksson, Anders Olofsson

**Affiliations:** 1 Department of Medical Biochemistry and Biophysics, Umeå University, Umeå, Sweden; 2 Department of Chemistry, Umeå University, Umeå, Sweden; 3 Airoptic Sp. z o.o. ZIWT, ul. Rubiez 46 H, 61–612, Poznan, Poland; National Center for Geriatrics and Gerontology, JAPAN

## Abstract

Transthyretin (TTR) is a homotetrameric plasma protein with amyloidogenic properties that has been linked to the development of familial amyloidotic polyneuropathy (FAP), familial amyloidotic cardiomyopathy, and senile systemic amyloidosis. The *in vivo* role of TTR is associated with transport of thyroxine hormone T4 and retinol-binding protein. Loss of the tetrameric integrity of TTR is a rate-limiting step in the process of TTR amyloid formation, and ligands with the ability to bind within the thyroxin binding site (TBS) can stabilize the tetramer, a feature that is currently used as a therapeutic approach for FAP. Several different flavonoids have recently been identified that impair amyloid formation. The flavonoid luteolin shows therapeutic potential with low incidence of unwanted side effects. In this work, we show that luteolin effectively attenuates the cytotoxic response to TTR in cultured neuronal cells and rescues the phenotype of a *Drosophila melanogaster* model of FAP. The plant-derived luteolin analogue cynaroside has a glucoside group in position 7 of the flavone A-ring and as opposed to luteolin is unable to stabilize TTR tetramers and thus prevents a cytotoxic effect. We generated high-resolution crystal-structures of both TTR wild type and the amyloidogenic mutant V30M in complex with luteolin. The results show that the A-ring of luteolin, in contrast to what was previously suggested, is buried within the TBS, consequently explaining the lack of activity from cynaroside. The flavonoids represent an interesting group of drug candidates for TTR amyloidosis. The present investigation shows the potential of luteolin as a stabilizer of TTR *in vivo*. We also show an alternative orientation of luteolin within the TBS which could represent a general mode of binding of flavonoids to TTR and is of importance concerning the future design of tetramer stabilizing drugs.

## Introduction

Transthyretin (TTR) is a well-defined homotetrameric serum protein composed of 127 amino acids. It is primarily produce by the liver, but expression of TTR is also found within the retina of the eye and within the brain. The function of TTR *in vivo* is to act as a transport protein for the thyroxine hormone T4 and the retinol-binding protein. Binding of the T4-hormone is achieved by two specific thyroxine binding sites (TBS) that are situated at the interface between the two TTR dimers.

TTR has amyloidogenic features and has been linked to the development of familial amyloidotic polyneuropathy (FAP), familial amyloidotic cardiomyopathy (FAC), and senile systemic amyloidosis [1]. In addition, TTR deposits have recently also been linked to the pathology of preeclampsia [2].

Both FAP and FAC are associated with mutations in TTR, and more than 100 unique pathological mutations have been identified [3]. These mutations frequently lower the stability of TTR and shift the equilibrium from a native state and may subsequently result in amyloid formation [4–6]. Although mutations frequently affect the thermodynamic stability of TTR, the rate-limiting step of TTR amyloid formation is dissociation of the tetramer [7, 8]. Liver transplantation is an effective treatment for FAP because this replaces the major source of mutated TTR production with the wild-type protein [1, 9]. However, a liver transplant is restricted only to mutated proteins and is moreover associated with additional risks linked to the surgical procedure, the subsequent immunosuppressive treatment, and also a continuous buildup of wild-type amyloid on pre-existing amyloid deposits [10, 11].

Alternative modes of treatment have been developed, including gene silencing [12] and the use of small molecules that prevent the dissociation of the TTR tetramer [8, 13–18]. Diflunisal and tafamidis (also known as Vyndaqel) have shown beneficial effects in clinical trials with FAP patients [19–21]. However, non-steroidal anti-inflammatory drugs (NSAID) like diflunisal are frequently associated with gastrointestinal side effects due to their inhibitory effect on the cyclooxygenase 1 system, and prolonged treatment may cause unwanted side-effects [22].

**Fig 1:**
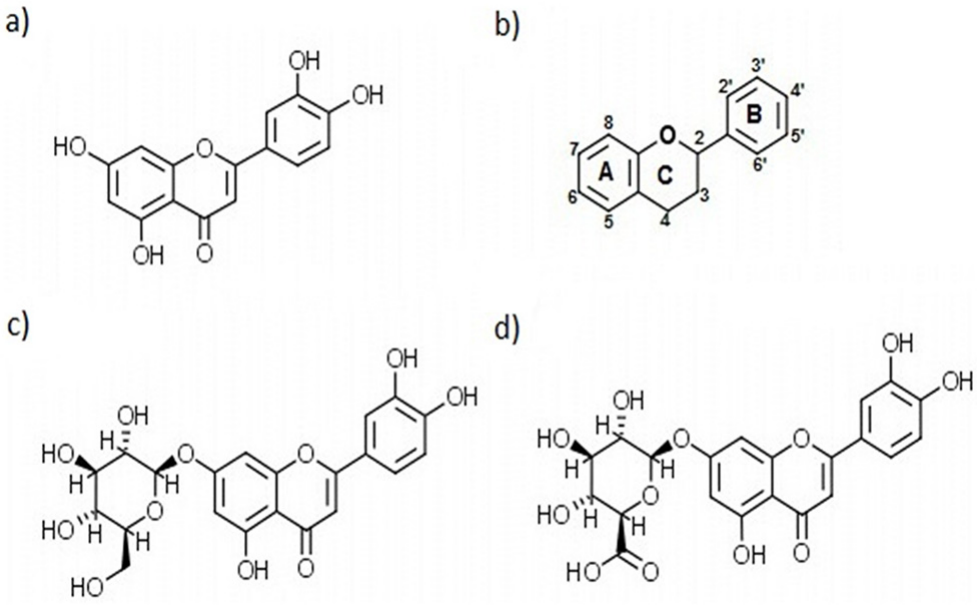
Flavonoids. (A) luteolin, (B) general formula of the flavonoid scaffold, (C) luteolin-7-O-glucoside, and (D) luteolin-7-O-glucuronide.

The ligand specificity of TTR is rather low, and several small organic molecules have been identified that have the potential to stabilize the tetrameric integrity of TTR [8, 13–18, 23]. Recently a group of flavonoids have been shown to bind and stabilize the TTR tetramer [24]. One of these—3´,4´,5,7-tetrahydroxyflavone (luteolin; Fig 1A)—is an abundant flavonoid produced in plants that has several health benefits, including neuroprotective properties [25, 26], and luteolin has recently been shown to prevent TTR aggregation *in vitro* [24]. Herbal extracts containing high doses of luteolin have been used for a long time in traditional Chinese medicine [27], and due to the rather low incidence of unwanted side effects, it is of interest to further investigate the ability of luteolin to stabilize the TTR tetramer and to prevent the toxic effects of tetramer dissociation. We show here that luteolin efficiently prevents the cytotoxic effects of TTR on a human neuroblastoma cell line and rescues the pathological phenotype in a *Drosophila melanogaster* model of FAP.

In humans, luteolin is subject to modification as a result of enzymatic activity within the liver and intestines. In particular position 7 of the A-ring, indicated in Fig 1B, is modified and is predominantly susceptible to glucuronidation as illustrated in Fig 1C. A recently published structure of TTR in complex with luteolin suggests that this position is solvent exposed [24], which suggests that modifications at position 7 is likely to be benign. However, we show here that a large modification at position 7 of the A-ring renders the luteolin molecule inactive. High-resolution crystal structures of native TTR and mutant TTRV30M in complex with luteolin show that luteolin is arranged with the A-ring buried within the core of the TBS, in agreement with the results of our *in vitro* activity assay.

Taken together, our experiments show how luteolin prevents TTR-induced pathologic effects both in cells and in an animal model for FAP. We present an alternative orientation of luteolin upon binding to the TBS of TTR that explains how glucuronidation would likely abrogate the effects of luteolin. The results may aid design of analogues with improved resistance to degradation in the quest to develop novel drugs for TTR-associated amyloid disorders.

## Results

### Luteolin prevents TTR toxicity in neuroblastoma cells

**Fig 2:**
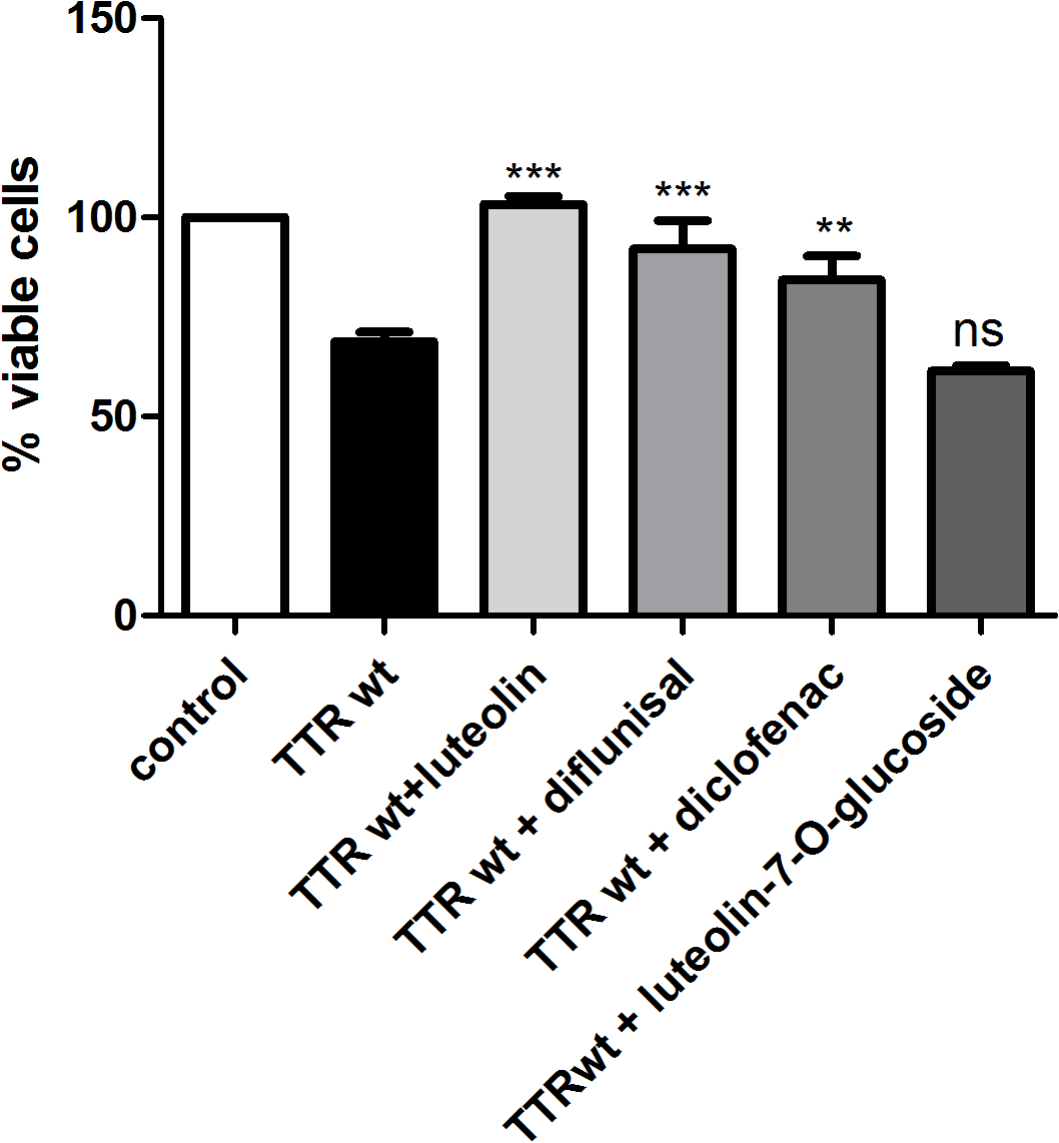
Inhibition of TTR-induced cytotoxicity. TTR at a final tetrameric concentration of 18 μM was pre-incubated with luteolin, diclofenac, diflunisal, or luteolin-7-*O*-glucoside (10 μM each) for 2 h. TTR or the TTR + inhibitor mixtures were added to SH-SY5Y cells and incubated for 72 h. Cell viability was measured with a resazurin assay [44]. Data are presented as mean ± standard deviation (n = 3, **P < 0.01; ***P < 0.001; ns P > 0.05). The addition of luteolin, diflunisal and diclofenac leads to a statistically significant difference in the number of viable cells compared to cells exposed to TTR alone, but the addition of luteolin-7-*O*-glucoside had no effect on TTR toxicity. The statistical significance was assessed using one-way ANOVA.

Luteolin has recently been found to bind to TTR and to prevent the dissociation of the tetramer [24], and the preservation of tetrameric integrity impairs the ability of TTR to exert a cytotoxic response [28]. In this work, we have used a cell-based viability assay to assess the ability of luteolin to suppress TTR-induced toxicity. The cytotoxicity of recombinant human TTR (TTR) in SH-SY5Y neuroblastoma cells has previously been demonstrated, and this was the experimental system used throughout the present investigation [29]. The cells were incubated with TTR at a concentration corresponding to 18 μM tetrameric TTR for a total time of 72 h. The response to TTR—as evaluated by a decrease in the conversion of resazurin to resorufin—was a 30% reduction in cell survival (Fig 2). Preincubation of TTR with 10 μM luteolin, diflunisal, or diclofenac increased the cell survival to essentially that of the untreated control cells (Fig 2).

### Luteolin reverses the phenotype of a *D*. *melanogaster* model of FAP

**Fig 3:**
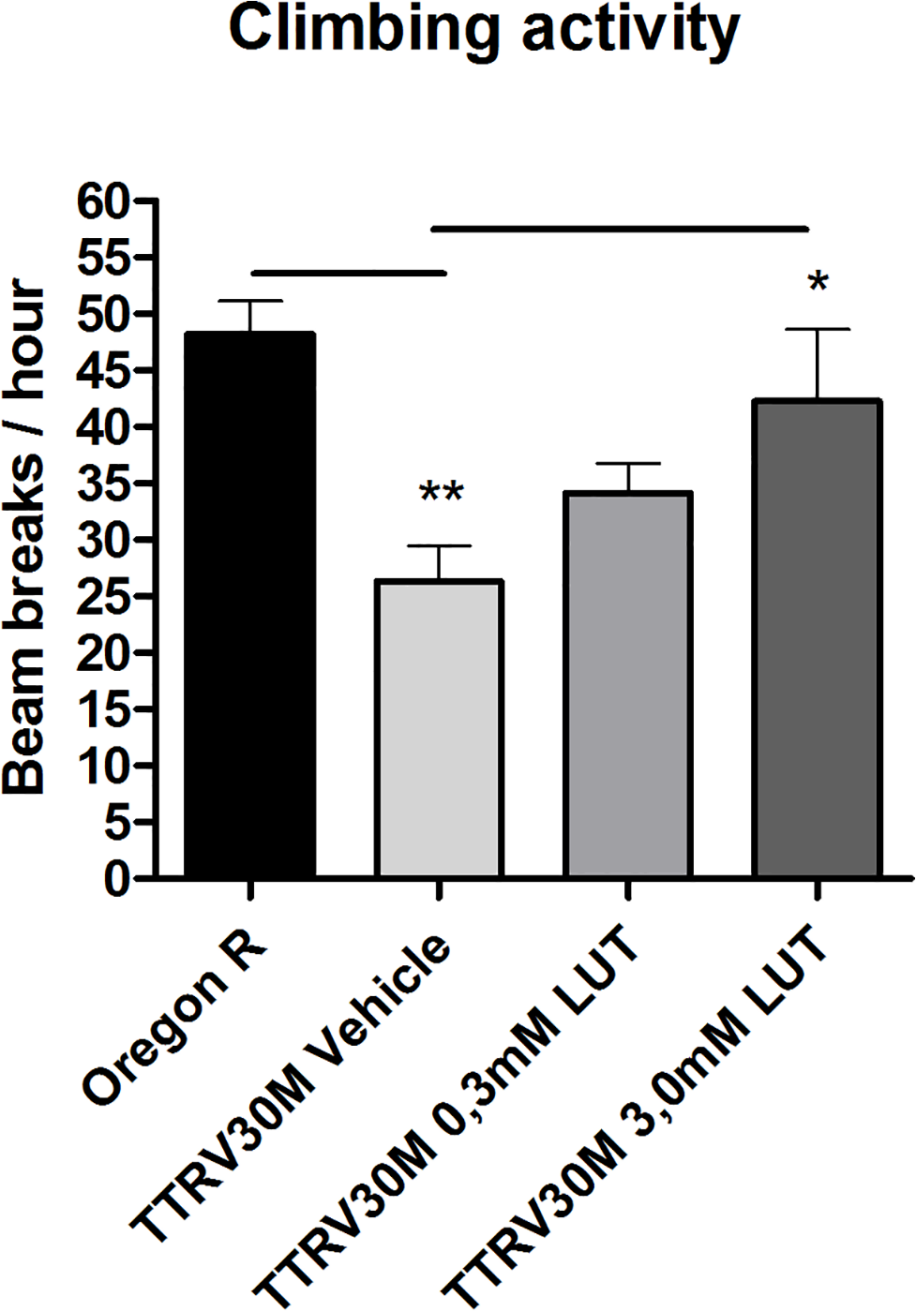
Luteolin reverses the phenotype of a *D*. *melanogaster* model of FAP. Climbing activity using a DAMS5 System is shown for wild-type flies and TTRV30M expressing flies without drug (vehicle) and with drug treatment at 6 days after eclosion. TTRV30M flies with vehicle alone showed reduced climbing activity compared to wild-type control flies. TTRV30M flies showed improved activity after drug treatment and a sufficient rescue effect was observed in flies treated with 3.0 mM luteolin. Values in graph represent mean ± S.E. *, *p* < 0.05 by Mann Whitney *U* test TTRV30M 3.0 mM LUT. **, *p* < 0.05 by Mann Whitney *U* test for TTRV30M vehicle.

To assess the ability of luteolin to mediate its stabilizing effect on TTR *in vivo* we have used an established animal model for FAP based on the fruit fly *D*. *melanogaster*. Flies expressing the mutated TTRV30M display an impaired neurological phenotype that resembles the clinical manifestations of TTR-associated amyloidosis in humans similarly to the published model with TTR V14N/V16E [30]. Luteolin was administered to the flies through their food. The food uptake in presence of luteolin was, in addition, verified to be normal using a standard blue dye food intake test. We used an established climbing assay [30] to evaluate the effect of luteolin and the result showed that luteolin rescued the impaired phenotype at 3 mM, illustrated in Fig 3.

### Luteolin-7-O-glucoside has no inhibitory effect on TTR aggregation

The activity of intestinal and liver enzymes in humans predominantly results in a high degree of conjugation of a glucuronide group at position 7 of luteolin. The general flavonoid scaffold is shown in Fig 1B while the specific structure of luteolin is shown in Fig 1A. Due to the potential use of luteolin as a kinetic stabilizer of TTR, it was of interest to evaluate the potential effect of luteolin after such a modification. Luteolin-7-*O*-glucuronide is not easily acquired, but luteolin-7-*O*-glucoside, also known as cynaroside, Fig 1C, is readily produced in plants and is highly similar in structure to luteolin-7-*O*-glucuronide and serves as a good analogue to investigate the effect of 7-*O*-glucuronidated luteolin shown in Fig 1D.

Using a well-established assay where the stability of TTR is monitored at low pH the effect of potential stabilizing drugs can be evaluated [17]. At a pH around 4.5 TTR is rapidly converted into opaque aggregates following a dissociation of the tetramer and a simple turbidity measurement can consequently be used to monitor changes in the kinetic stability.

**Fig 4:**
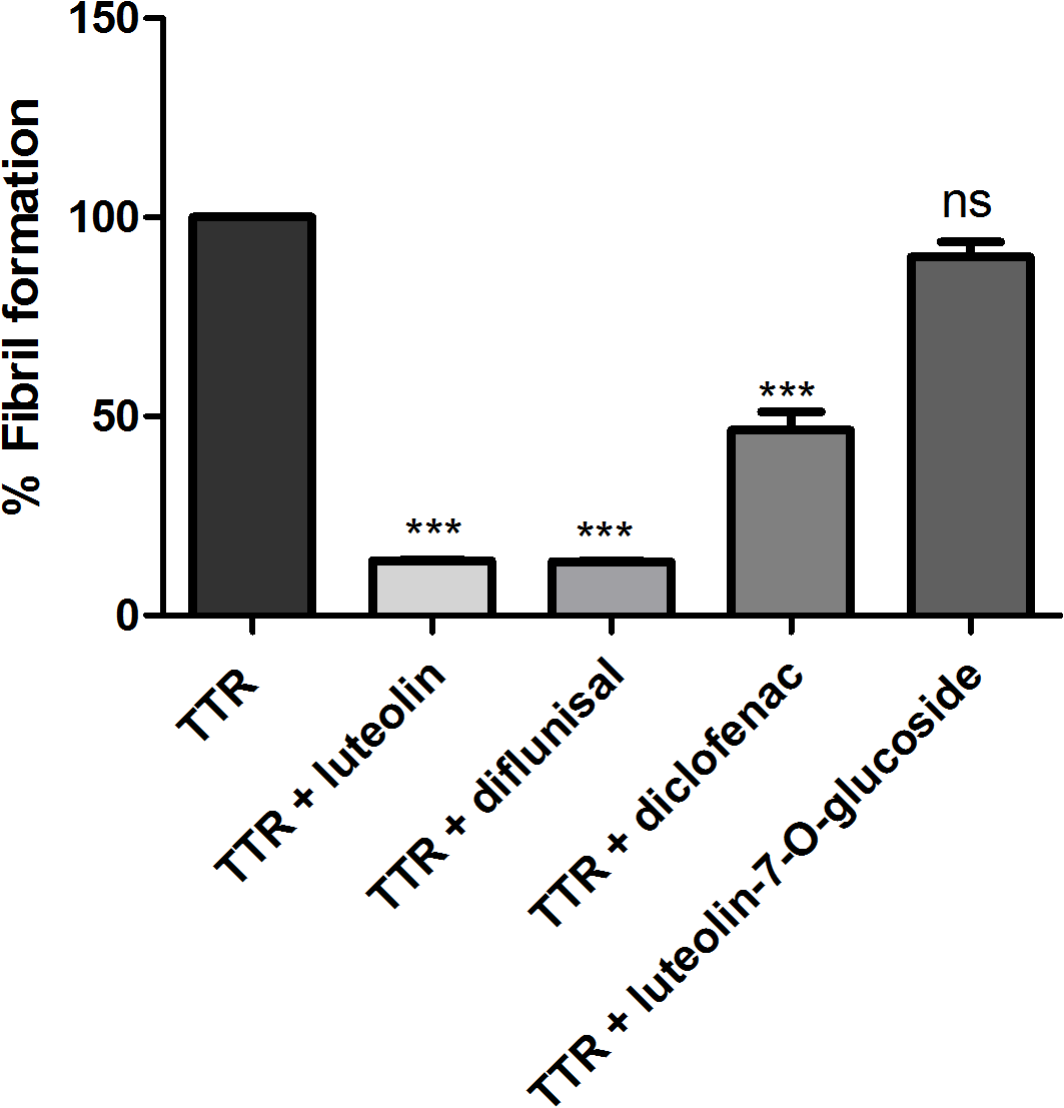
Influence of inhibitors on TTR aggregation under acidic conditions. TTR (final tetrameric concentration 15 μM) was pre-incubated with luteolin, diflunisal, diclofenac, or luteolin-7-*O*-glucoside (each at 15 μM) for 2 h. The TTR complexes were subjected to pH 4.6 for 72 h, and fibril formation was evaluated by turbidity measurements at 400 nm [17]. The amount of aggregated TTR is reported as a fraction of fibril formation relative to the amount of aggregates produced in the absence of inhibitors. There was a significant decrease in TTR fibril formation after the addition of luteolin, diflunisal, and diclofenac compared with TTR alone, but no inhibitory effect was observed after treatment with luteolin-7-*O*-glucoside. The statistical significance of the obtained results was assessed using one-way ANOVA. Data are presented as mean ± standard deviation of the % fibril formation (n = 2 ***P < 0.001; ns P > 0.05).

The results show that a glucoside group in position 7 of the A-ring of luteolin abolishes its ability to inhibit pH induced fibril formation (Fig 4). This is in accordance with luteolin-7-*O*-glucoside’s inability to rescue cell-viability, as shown previously in Fig 2.

### The aromatic A-ring of luteolin is buried within the TBS of TTR

**Fig 5:**
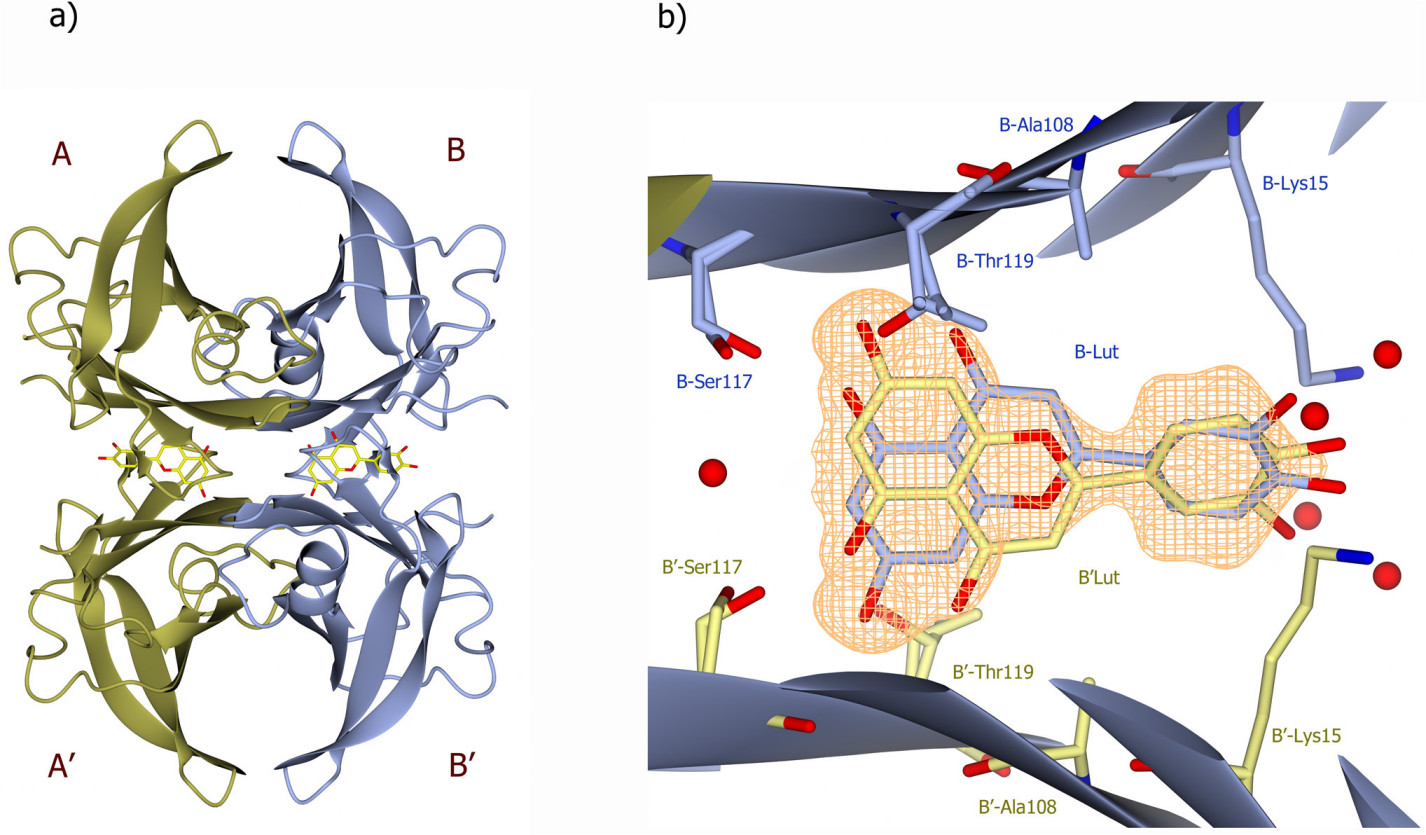
The crystal structure of TTR-luteolin complex. A) The TTR monomers in the dimer structure are shown as ribbons and are labeled A and B. The symmetry-related monomers are labeled A´, and B´. Two luteolin molecules bind at the thyroxin-binding channels, and are shown as sticks. For clarity, only one of the symmetry-related luteolin orientations is shown. B) The quality of the electron density map at the BB´ dimer-dimer interface. The σA-weighted (m|Fo|-D|Fc|) electron density contoured at 3 times the root-mean-square value of the map is shown in orange. To reduce model bias the luteolin molecule was excluded from the coordinate file that was subjected to one round of simulated annealing refinement before calculation of the map.

**Fig 6:**
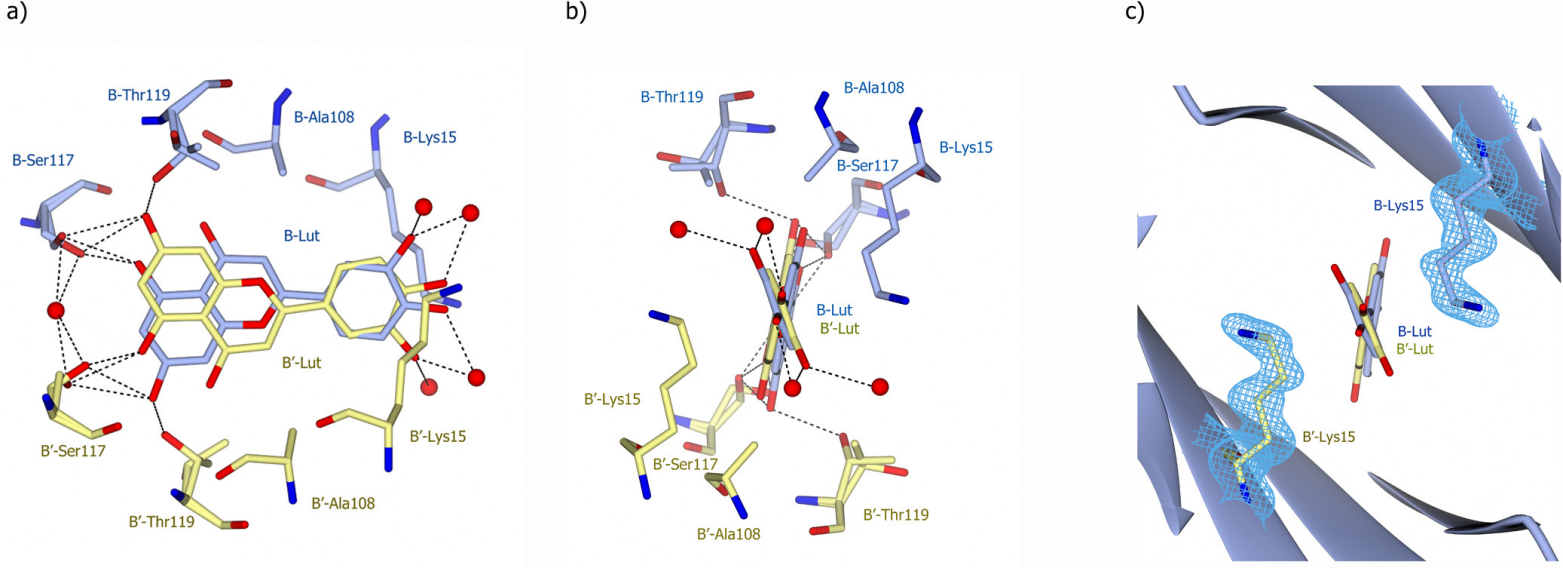
Detailed view of the luteolin-binding site in TTR. A and B) Two orientations of the TTR-luteolin complex that show interactions made between the luteolin A-ring and TTR. Residues and the luteolin molecule from the B and B’ monomers are shown in blue and yellow, respectively. Hydrogen bonds are shown as dotted lines. The O_5_ and O_7_ oxygen form hydrogen bonds to the Oγ1 atoms of B-Ser117 and B-Thr119 (and B´-Ser117 and B’-Thr119 over the tetramer interface). Both Ser117 and Thr119 are refined in two conformations. C) The σ^A^-weighted (2m|Fo|-D|Fc|) electron density calculated from the refined coordinates is contoured at the root-mean-square value of the mapover residues B- and B’-Lys15 (blue). The density shows that no direct hydrogen bond is formed between the Nz atom of Lys15 to luteolin.

Crystal structures of TTR-luteolin and TTRV30M-luteolin complexes were determined at 1.12 and 1.7 Å resolution, respectively, S1 Table. The TTR-luteolin complexes were co-crystallized with five molar excess of inhibitor in the space group P2_1_2_1_2 and contains one dimer in the asymmetric unit. By applying the crystallographic 2-fold symmetry at the c-axis the biological dimer could be obtained (Fig 5A). The inner β-sheets of the dimer–dimer (AB–A´B´) interface form two ligand-binding cavities (TBSs) referred to as sites AA´ and BB´. Specific high-affinity binding of luteolin to TTR and V30M were confirmed from difference Fourier electron density maps, Fig 5B and S1 Fig. The TTR-luteolin tetramer is structurally very similar to TTR [31]. Each monomer in the structure consists of residues Cys10-Pro125 as previously reported. Luteolin binds in the hydrophobic TBS. The electron density of the complex structure suggest that the orientation of the ligand should be rotated 180° in comparison with the previously reported TTR-luteolin structure (pdb code 4dew, [24]). The O_5_ and O_7_ atoms positioned on the A-ring of luteolin form in the new orientation hydrogen bonds with the side chains of Ser117, Thr119 and symmetry-related copies of the same residues over the dimer-dimer interface (Fig 6A and 6B). No additional hydrogen bonds are formed between the protein and the luteolin ligand. Interactions between the luteolin B-ring and TTR involve van der Waal contact to the side chain of Leu17 and the Cδ and Cε atoms of Lys15. The side chain of Lys15 is directed away from luteolin oxygens O_3´_ and O_4´_ at both binding pockets (Fig 6C). The latter luteolin oxygen, positioned on the B-ring, make hydrogen bonds with water molecules positioned at the surface of the molecule (Fig 6A and 6B). The water molecules hydrogen bonded to the B-ring are however not so well defined in the electron density, which indicate that they are flexible. The TTRV30M-luteolin tetramer is structurally very similar to TTR-luteolin, and shows the same orientation of luteolin at the TBS, S1 Fig.

## Discussion

The neuropathological properties of TTR are puzzling, and it is not yet fully understood how it exerts its toxic effect. However, regardless of the downstream steps leading to the formation of cytotoxic species, it is clear that a primary target for interventions is the dissociation of the TTR tetramers [6]. In other words, preventing the initial step of tetrameric dissociation will also prevent any subsequent conversion into pathological structures. The properties of TTR can be divided into a thermodynamic component that represent the equilibrium between the folded and unfolded state at equilibrium and a kinetic component that defines the rate at which the structure unfolds under denaturing conditions [32, 33]. Dissociation of the tetramer is rate limiting and is modulated by ligand binding. Although a strong effect can be seen upon binding of thyroxin hormone T4 [34], the levels of thyroxine hormone T4 in human serum only binds approximately 1% of total TTR and it is not feasible to increase these levels due to adverse side effects. Therefore, alternative ligands have been presented [13, 14, 17, 35–40] and a few have proven to be beneficial from a therapeutic point of view [19, 20]. Recently a group of flavonoids have been shown to prevent dissociation of the tetramer, and luteolin demonstrated particularly potent inhibitory properties of TTR and TTRV30M [24]. Luteolin has several interesting physiological properties and has been shown to be beneficial in protecting against neuronal damage [25, 26]. Luteolin is widely used within traditional Chinese medicine and has few side effects even upon prolonged intake at high doses [27]. These properties make luteolin an interesting candidate for the potential treatment of TTR-induced amyloidosis in humans.

In this work, we have shown that luteolin prevents the cytotoxic effect of TTR on a human neuronal cell line and that it also rescuers the pathological phenotype in a *D*. *melanogaster* model of FAP, which suggest that luteolin can exert its effects within a complex environment. The TTRV30M phenotype shows essentially no TTR immunoreactive aggregates in the fly, in contrast to the previously reported Drosophila variant TTR V14N/V16E [41]. This suggests that the toxic effect does not correlate well with the total load of aggregates. Such result is further supported by cell studies, where the stability of a tetramer instead correlates with toxic effect [29, 42]. Measuring the stability of the TTR tetramer within the fly is however technically challenging and could not be performed.

In humans luteolin is unfortunately subjected to modification by enzymes produced in the liver and intestines which results primarily in the addition of a glucuronide modification to the hydroxyl group in position 7 of the A-ring [43]. In a recent crystal structure of TTR in complex with luteolin, the orientation of the luteolin showed position 7 of the A-ring to be solvent exposed, and this suggests that modifications at this position would have none or just a small effect on luteolin’s interactions with TTR [24]. Evaluation of the effect of a modification at position 7 can easily be tested using a well-established turbidity assay based on low pH [17]. However, luteolin-7-*O*-glucuronide cannot be purchased and we have therefore evaluated the effect of the highly similar luteolin-7-*O*-glucoside that is readily produced in plants (see Fig 1 for a structural comparison). Given the hypothesis that this position would be solvent exposed, subtle variations in the conjugated moiety would not be expected to interfere with luteolin binding to TTR. We found, however, that luteolin-7-*O*-glucoside is inactive. To investigate the molecular interaction between luteolin and TTR in more detail, we solved an atomic-resolution crystal structure of a complex between TTR and luteolin.

Our structure showed that luteolin was oriented such that the A-ring rather than the B-ring was facing the core of the TBS (Fig 5B). This orientation fits well with our biochemical data as the bulky addition at position 7 of luteolin-7-*O*-glucoside prevents it from binding at TBS and explains the lack of activity. This orientation of luteolin was also verified in the TTRV30M- luteolin complex structure, S1 Fig. We hypothesize that a similar orientation would be seen for all other flavonoids that are reported to bind TTR.

From a perspective of drug-design, the present result suggests that replacing the hydroxyl group in position 7 of luteolin with a less reactive group or atom e.g. using a halide or a small alkyl group might preserve its binding properties to TTR while at the same time increase its stability *in vivo*.

Taken together, our results show how luteolin specifically prevents the cytotoxic effect of TTR both in cell culture experiments and in a *D*. *melanogaster* model of FAP. We also show that the luteolin molecule binds to the TBS with its aromatic A-ring buried within the TBS and the B-ring facing the solvent. This mode of interaction is likely to be representative of the entire group of flavonoids and explains how bulky modifications of the hydroxyl group located in position 7 of the luteolin scaffold will impair its effect. Due to its efficacy in the *D*. *melanogaster* model for FAP, as well as its beneficial properties against neuronal damage in humans, luteolin and potentially glucurunoidation-resistant analogues, where the hydroxyl group in position 7 has been substituted, are interesting candidates in the quest to develop novel interventions for the treatment of TTR-associated disorders.

## Experimental Section

### Recombinant expression and purification of TTR

Expression of TTR was performed according to a previously published protocol [29]. Briefly, after transformation of the plasmid into *Escherichia coli* BL21, the cells were grown until an OD_600_ of 0.6 followed by induction with 0.4 mM isopropyl thiogalactopyranoside at 37°C. After 18 hours, cells were harvested and lysed by sonication. Cell debris was removed by centrifugation at 20,000 × *g* for 30 min, and the supernatant was collected and loaded onto an anion exchange column (Q-sepharose, Amersham Biosciences) and eluted with a NaCl gradient. The fractions containing TTR were concentrated using Centriprep filter units with Ultracel YM-10 membranes (Millipore) and loaded onto a gel filtration column (Superdex G75-16/60, Amersham Biosciences) equilibrated in minimum essential medium (MEM). The purified protein solution was supplemented with 2 mM L-glutamine, 100 units/mL penicillin, 100 μg/mL streptomycin (Gibco), and 1% non-essential amino acid solution before incubation with SH-SY5Y cells.

### Cell culture

Neuroblastoma SH-SY5Y cells were obtained from the European Collection of Cell Cultures (Center for Applied Microbiology and Research). Cells were cultured in MEM and GlutaMax (Gibco) and supplemented with 10% (v/v) fetal bovine serum (Gibco), 100 units/mL penicillin, 100 μg/mL streptomycin (Gibco), and 1% non-essential amino acid solution (Gibco). Cultures were maintained in an incubator at 37°C with a humidified atmosphere of 5% CO_2_.

### Inhibition of TTR-induced cytotoxicity

Luteolin, diflunisal, diclofenac, and luteolin-7-*O*-glucoside were pre-incubated with purified TTR in MEM with supplements for two hours before being added to the SH-SY5Y cells. The TTR was added to the cells at a final concentration of 18 μM while all inhibitors were applied at 10 μM. The mixtures were incubated with the cells for 48 h at 37°C in a humidified atmosphere of 5% CO_2_. Cytotoxicity was evaluated using a resazurin reduction test [44] and the viability of the cells was detected by fluorescence measurement using a Tecan Safire plate reader with excitation at 535 nm and emission at 595 nm. All experiments were performed in triplicate.

### Influence of inhibitors on TTR aggregation under acidic denaturing conditions

TTR at a final concentration of 15 μM was pre-incubated with luteolin, diflunisal, diclofenac, or luteolin-7-*O*-glucoside (15 μM each) for 2 hours at room temperature. Acidic denaturation (pH = 4.6) was achieved by adding acetate buffer and incubating for 72 h. The formation of TTR aggregates was detected by turbidity measurement of the OD_400_ using a Tecan Safire plate reader.

### *D*. *melanogaster* model of FAP

UAS-TTR V30M flies (generated as described in [30]) were expressed under control of the pan-neuronal n-syb-GAL4 promoter, which was a kind gift of Dr. Julie Simpson (Howard Hughes Medical Institute, Chevy Chase, MD). Wild-type Oregon-R strain flies were originally obtained from the Bloomington stock center and crossed with the n-syb-GAL4 in control experiments.

All inhibitors were dissolved in 95% ethanol and mixed into the standard corn/yeast/agar fly food at appropriate concentrations. The final ethanol concentration in the food corresponded to 4.8% (w/v) in both treated and controls. Flies were given access to the drugs *ad libitum* during their development and for one week after their eclosion. Standard fly food was mixed with blue food-dye (Patent Blue V) at concentration 0.5 mg/ml as internal marker of food intake, S2 Fig. This food additionally contained vehicle or tested compounds at indicated concentrations. Fresh food was provided every second day and flies were allowed to feed for one week starting from larval stages. The blue food-dye used, at the above concentration, was ascertained to be neither toxic nor was it metabolized. After one week of rearing under standard procedures, flies were anesthetized, placed under a stereomicroscope (SMZ-140/143, Motic, Wetzlar, Germany) and imaged using a digital camera (Olympus SC30, 3.1 MP, Hamburg, Germany) with Olympus Stream Essentials software. Food intake was quantified as blue food-dye content in the gut and eating ability of flies was not affected.

Flies were reared at 25°C under 12 h cycles of light and darkness. Climbing activity of female flies was monitored using the Drosophila Activity Monitoring System (DAMS5) (Trikinetics, Waltham, MA). Activity counts were collected in 1 min bins over 24 hours and are presented as the average number of beam breaks per hour. Graphs and statistical comparisons were generated with IBM SPSS 20 Statistics (IBM Corporation, Armonk, NY). The Mann Whitney *U* test was used for comparing treatment-group means.

### Crystallization of the TTR-luteolin complex and TTRV30M-luteolin complexes

The protein was crystallized as described previously [45]. Briefly, the purified TTR was dialyzed against 10 mM Na-phosphate buffer with 100 mM KCl (pH 7.6) and concentrated to 5 mg·ml^−1^ using an Amicon Ultra centrifugal filter device (Millipore, 3 kDa molecular-weight cutoff) and co-crystallized at room temperature with a 5 molar excess of luteolin using the vapor-diffusion hanging drop method. A drop containing 3 μL protein solution was mixed with 3 μL precipitant and equilibrated against 1 mL reservoir solution containing a range of 1.3–1.6 M sodium citrate and 3.5% *v/v* glycerol at pH 5.5 in 24-well Linbro plates. Crystals grew to dimensions of 0.1 × 0.1 × 0.4 mm^3^ after 5 days. The crystals were cryoprotected with 12% *v/v* glycerol.

### Data collection, integration and structure determination

The X-ray diffraction data of the TTR-luteolin complex were collected under cryogenic conditions to 1.12 Å resolution at the European Synchrotron Radiation Facility in Grenoble, France, on beamline ID23-1 using Pilatus 6M detectors at a wavelength of 0.900 Å. Data were collected with a crystal-to-detector distance of 184.4 mm and 0.15° oscillations per image. The diffraction data were processed with XDS [46] and scales using SCALA from the CCP4 software suite [47]. The X-ray model of TTR (pdb code 1F41, [31]) and X-ray data from 38.2–2.5 Å resolution were used in molecular replacement searches with the program MOLREP [48]. The luteolin molecule is best defined in the BB´ cavity. The model was initially refined against all the diffraction data using REFMAC [49]. Manual map inspections was performed with COOT [50]. During refinement isotropic B-factors were first applied followed by anisotropic B-factors at the end of the refinement. At the end of structure refinement, phenix.refine from the PHENIX program suite [51] was used to perform successive cycles of translation/libration/screw (TLS) refinement resulting in an *R* factor of 13.8% and an *R*_free_ of 15.3% using all data in the range 38.2 Å–1.12 Å. The refined structure comprises residues 10–125 of TTR, 2 luteolin molecules, 3 glycerol molecules, and 271 water molecules. There are no Ramachandran outliers. The loop regions Tyr78-Ser85 in monomer A and Asp99-Arg104 in monomer B are modelled in two conformations. Molecular graphics were produced using CCP4mg [52].

The TTRV30M-luteolin complex was solved and refined essentially as described for the TTR-luteolin complex. Diffraction data were collected on our in-house Bruker MicroStar facility at 1.7 Å resolution, and were processed and scaled with SAINT and SCALA. The structure was refined with isotropic B-factors only. See S1 Table for details of data collection and refinement statistics.

Structure factors and coordinates of the TTR-luteolin and TTRV30M-luteolin complexes have been deposited at the protein data bank (accession codes: 4qxv and 4qya, respectively).

## Supporting Information

S1 Fig The quality of the electron density at the BB´ dimer-dimer interface of the TTRV30M-luteolin complex. The σ^A^-weighted (m|Fo|-D|Fc|) electron density is contoured at 3 times the root-mean-square value of the map and shown in orange. To reduce model bias the luteolin molecule was excluded from the coordinate file that was subjected to one round of simulated annealing before calculation of the electron density map. The orientation of luteolin in the V30M mutant is identical to the one observed in the TTRwt-luteolin complex.
(TIFF)

S2 Fig Food uptake by flies. Standard fly food was mixed with blue food-dye at concentration 0.5 mg/ml as internal marker of food intake. Fresh food was provided every second day and flies were allowed to feed for one week starting from larval stages. After one week of rearing under standard procedures, flies were anesthetized, placed under a stereomicroscope and imaged using a digital camera with Olympus Stream Essentials software. Food intake was quantified as blue food-dye content in the gut.
(TIF)

S1 Table X-ray data collection and refinement statistics for TTRwt-luteolin and TTRV30M complexes. (DOCX)
