# Supplementary figures and images for: The Flavonoid Luteolin, but Not Luteolin-7-O-Glucoside, Prevents a Transthyretin Mediated Toxic Response
Source: PLoS One. 2015 May 28;10(5):e0128222. doi: 10.1371/journal.pone.0128222 (PMC4447256; doi:10.1371/journal.pone.0128222)

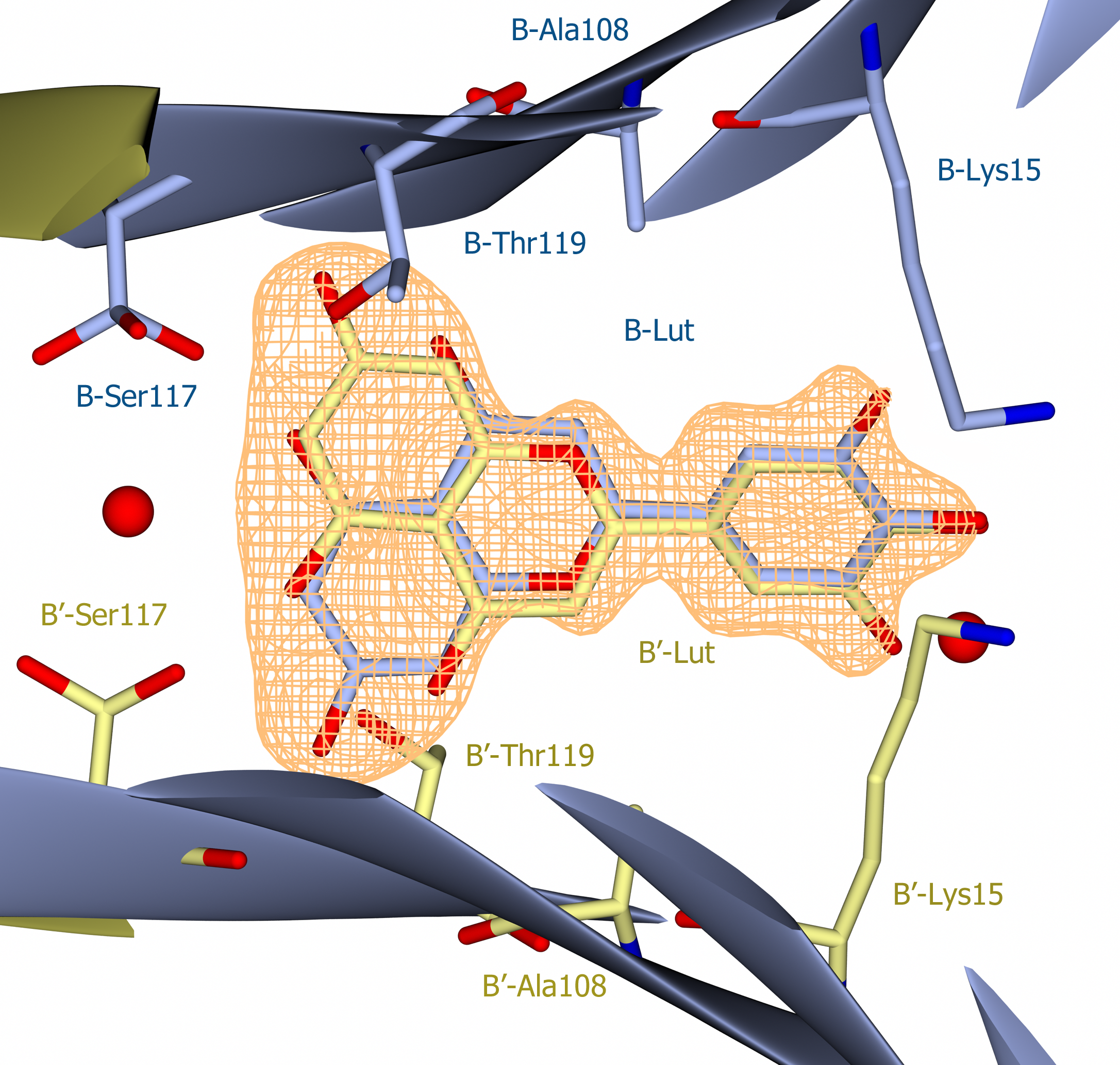

Supplement: S1 Fig — The σA-weighted (m|Fo|-D|Fc|) electron density is contoured at 3 times the root-mean-square value of the map and shown in orange. To reduce model bias the luteolin molecule was excluded from the coordinate file that was subjected to one round of simulated annealing before calculation of the electron density map. The orientation of luteolin in the V30M mutant is identical to the one observed in the TTRwt-luteolin complex. (TIFF) [file pone.0128222.s001.tiff]

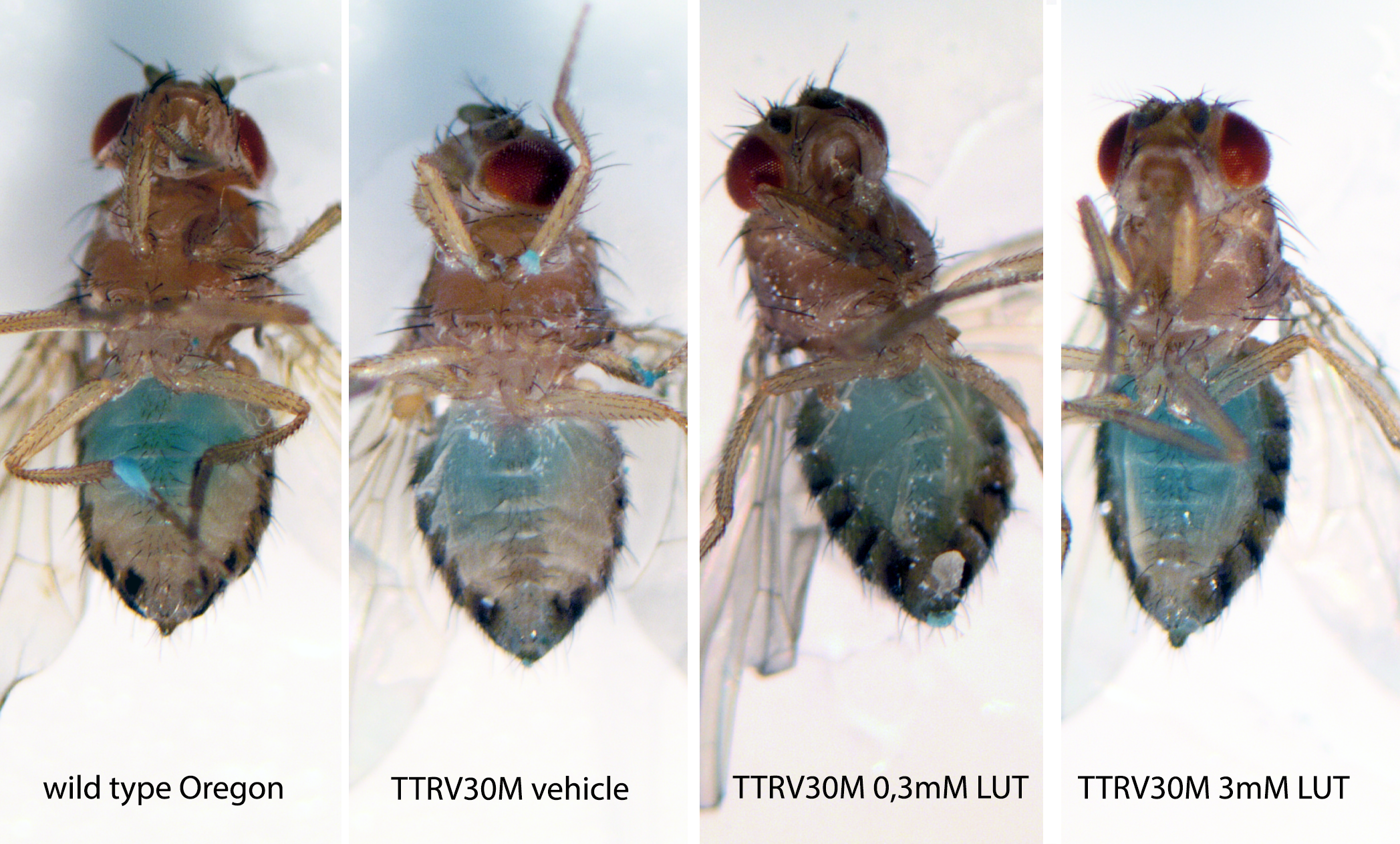

Supplement: S2 Fig — Standard fly food was mixed with blue food-dye at concentration 0.5 mg/ml as internal marker of food intake. Fresh food was provided every second day and flies were allowed to feed for one week starting from larval stages. After one week of rearing under standard procedures, flies were anesthetized, placed under a stereomicroscope and imaged using a digital camera with Olympus Stream Essentials software. Food intake was quantified as blue food-dye content in the gut. (TIF) [file pone.0128222.s002.tif]
