# Supplementary material for: The Flavonoid Luteolin, but Not Luteolin-7-O-Glucoside, Prevents a Transthyretin Mediated Toxic Response
Source: PLoS One. 2015 May 28;10(5):e0128222. doi: 10.1371/journal.pone.0128222 (PMC4447256; doi:10.1371/journal.pone.0128222)
Supplement: S1 Table — (DOCX) [file pone.0128222.s003.docx]

**Supporting Information**

The Flavonoid Luteolin, but not Luteolin-7-*O*-glucoside, Prevents a Transthyretin Mediated Toxic Response

Irina Iakovleva^1*^, Afshan Begum^2*^, Malgorzata Pokrzywa^3^, Malin Walfridsson^1^, A. Elisabeth Sauer-Eriksson^2^ and Anders Olofsson^1^

^1^Department of Medical Biochemistry and Biophysics, Umeå University, 901 87 Umeå, Sweden

^2^Department of Chemistry, Umeå University, 901 87 Umeå, Sweden.

^*^Both authors contributed equally to this work.

^3^Airoptic Sp. z o.o. ZIWT, ul. Rubiez 46 H, 61-612, Poznan, Poland

Correspondence: anders.olofsson@medchem.umu.se

| **Data-collection parameters** | **TTRwt-luteolin** | **TTRV30M-luteolin** |
| --- | --- | --- |
| Wavelength (Å) | 0.9002 | 1.5400 |
| Temperature (K) | 100 | 100 |
| Oscillation range (°) | 0.15 | 0.5 |
| Crystal-to-detector distance (mm) | 184.4 | 60 |
| **Data-integration statistics** |  |  |
| Space group | P21212 | P21212 |
| Unit-cell parameters (Å) | a = 42.6, b = 85.9, c = 63.9 | a = 42.8, b = 85.8, c = 63.7 |
| Resolution limits (Å ) | 38.2-1.12 (1.16 - 1.12) | 23.81 - 1.70 (1.76 - 1.70) |
| Total No. of reflections | 524231 | 228831 |
| No. of unique reflections | 91106 (8981) | 26524 (2500) |
| Multiplicity | 5.7(5.5) | 8.6 (8.4) |
| Completeness (%) | 99.8 (99.9) | 99.59 (95.93) |
| R_merge_† | 0.047 (0.36) | 0.045 (0.31) |
| Mean I/(σ (I) | 17.29 (3.49) | 43.31 (6.07) |
| **Refinement and model building statistics** | | |
| Resolution Range (Å ) | 38-1.12 | 23.81 - 1.70 |
| R factor (%) | 13.6(17.2) | 16.2(21.0) |
| R free (%) | 15.1(17.6) | 19.7(24.0) |
| No. of protein atoms | 2298 | 2205 |
| No. of water atoms | 250 | 271 |
| No. of ligand/ion | 4/1 | 2/1 |
| **R.m.s. deviations from ideal geometry** |  |  |
| Bond distances (Å ) | 0.012 | 0.007 |
| Bond angles (^o^) | 1.7 | 1.10 |
| Ramachandran plot |  |  |
| Residues in most favored regions (%) | 98.04 | 98.05 |
| Residues in additional allowed regions (%) | 1.57 | 2.0 |
| Residues in disallowed regions (%) | 0.0 | 0.0 |
| Average B-factor (Å^2^) | 18.2 | 19.8 |
|  |  |  |
| †Rmerge = ∑hkl ∑i \| Ii {hkl} – {I (hkl)} \| ⁄ ∑hkl∑i Ii (hkl), where {I (hkl)} is the mean intensity of the observations Ii (hkl) of reﬂection hkl. R_free_ is based upon 5% of the data randomly culled and not used in the refinement. | | |
